# Effect of Two Different Multimicronutrient Supplements on Vitamin D Status in Women of Childbearing Age: A Randomized Trial

**DOI:** 10.3390/nu9010030

**Published:** 2017-01-04

**Authors:** Stefan Pilz, Andreas Hahn, Christiane Schön, Manfred Wilhelm, Rima Obeid

**Affiliations:** 1 Division of Endocrinology and Diabetology, Department of Internal Medicine, Medical University of Graz, Auenbruggerplatz 15, 8036 Graz, Austria; 2 Institute of Food Science and Human Nutrition, Leibniz University of Hannover, Am Kleinen Felde 30, 30167 Hannover, Germany; hahn@nutrition.uni-hannover.de; 3 BioTeSys GmbH, Schelztorstrasse 54-56, 73728 Esslingen, Germany; c.schoen@biotesys.de; 4 Department of Mathematics, Natural and Economic Sciences, University of Applied Science Ulm, Albert-Einstein-Allee 55, 89081 Ulm, Germany; wilhelm@hs-ulm.de; 5 Aarhus Institute of Advanced Studies, University of Aarhus, Hoegh-Guldbergs Gade 6B, Building 1632, DK-8000 Aarhus, Denmark; rima.obeid@uks.eu

**Keywords:** randomized controlled trial, vitamin D, supplementation, multimicronutrient, women, 25(OH)D

## Abstract

The German Nutrition Society raised in 2012 the recommended daily vitamin D intake from 200 to 800 international units (IU) to achieve 25-hydroxyvitamin D (25(OH)D) levels of at least 50 nmol/L, even when endogenous vitamin D synthesis is minimal such as in winter. We aimed to evaluate this recommendation in women of childbearing age. This is a single-center, randomized, open trial conducted from 8 January to 9 May 2016 in Esslingen, Germany. We randomized 201 apparently healthy women to receive for 8 weeks a daily multimicronutrient supplement containing either 200 IU (*n* = 100) or 800 IU vitamin D3 (*n* = 101). Primary outcome measure was serum 25(OH)D. 196 participants completed the trial. Increases in 25(OH)D (median with interquartile range) from baseline to study end were 13.2 (5.9 to 20.7) nmol/L in the 200 IU group, and 35.8 (18.2 to 52.8) nmol/L in the 800 IU group (*p* < 0.001 for the between group difference). At study end, levels of ≥50 nmol/L were present in 70.4% of the 200 IU group and in 99% of the 800 IU group. Participants on hormonal contraceptives had higher baseline levels and a stronger increase in 25(OH)D. In conclusion, daily supplementation of 800 IU vitamin D3 during wintertime in Germany is sufficient to achieve a 25(OH)D level of at least 50 nmol/L in almost all women of childbearing age, whereas 200 IU are insufficient.

## 1. Introduction

Vitamin D is classically known for its role in bone and mineral metabolism, but low levels of 25-hydroxyvitamin D (25(OH)D), the vitamin D metabolite that is used to assess vitamin D status, have also been associated with various extra-skeletal diseases such as cancer, infections and cardiovascular diseases [1,2,3,4]. While there is an ongoing scientific debate on the cause and effect relationship of vitamin D deficiency with various acute and chronic diseases, nutrition societies have almost universally accepted that vitamin D is required for maintenance of skeletal health, in particular for the prevention of rickets and osteomalacia [5,6,7,8,9]. It is therefore of public health concern that vitamin D deficiency is common in the general population. A European survey documented that 13.0% of the population have 25(OH)D levels below 30 nmol/L (divide by 2.496 to convert nmol/L to ng/mL) and 40.4% below 50 nmol/L [10]. Considering that ultraviolet-B (UV-B)-induced vitamin D synthesis in the skin is usually the major source of vitamin D in humans, whereas nutrition plays only a minor role, it has been observed that 25(OH)D levels are significantly lower in winter compared to summer [10]. In most European countries including Germany or in northern regions of the US, the UV-B radiation is too weak during the winter months to induce adequate vitamin D synthesis in the skin [11]. Therefore, there is a need to ensure a sufficient vitamin D intake during winter because the half-life of 25(OH)D serum levels is only about 2 to 3 weeks so that even individuals with high 25(OH)D levels in summer are at risk of vitamin D deficiency in winter.

The US Institute of Medicine (IOM) adopted its vitamin D recommendations in 2010 and estimated that 25(OH)D levels of at least 50 nmol/L would meet the vitamin D requirements of 97.5% of the population although there is still a debate on the optimal levels with the recommendation of the Endocrine Society to aim for 25(OH)D levels of >75 nmol/L [5,6]. According to the IOM, the recommended dietary allowance (RDA) to meet the nutritional requirements for vitamin D in 97.5% of the population is 600 (age 1 to 70 years) to 800 international units (IU) (70 years or older) vitamin D per day (40 IU is equal to 1 µg vitamin D) [5]. These estimates were based on meta-regression analyses of randomized controlled trials (RCTs) in winter on the dose-response of vitamin D intake and serum 25(OH)D levels [5]. In 2012, the Nutrition Societies in Germany, Austria and Switzerland (DACH) published new vitamin D recommendations and considered, in line with the IOM report, 25(OH)D levels of 50 nmol/L or higher as an indicator of optimal vitamin D status [12]. The previous intake recommendation of 200 IU per day was raised to 800 IU per day and applies to all individuals aged 1 year or older, and to conditions when endogenous vitamin D synthesis is missing [12]. This recommendation was based on Irish studies by Cashman et al. who showed that during wintertime, a vitamin D intake of 800 IU per day is sufficient to achieve a 25(OH)D level of ≥50 nmol/L in about 90% to 95% of the Irish population [12,13].

More RCTs on the dose response relationship of vitamin D in the general population were published since the DACH Nutrition Society published its new guideline [14,15,16,17,18]. However, there is, to the best of our knowledge, no randomized trial available comparing the old (200 IU) versus the new (800 IU) vitamin D intake recommendations in the general population in Germany. We therefore aimed to address this knowledge gap in a randomized trial in women of childbearing age. In such a population, vitamin D may, beyond its role in bone health, be of particular importance because the unborn child is dependent on the mother’s 25(OH)D levels, and vitamin D deficiency has been associated with various adverse health outcomes in pregnancy [19,20,21,22,23]. Given that vitamin D status may be modified by intake of hormonal contraceptives, we also evaluated the impact of hormonal contraceptive use on 25(OH)D levels and their increase after vitamin D supplementation [24,25]. Our trial was, however, not designed to evaluate effects of vitamin D supplementation on specific diseases or to address the question which 25(OH)D levels are optimal for disease prevention.

## 2. Materials and Methods

### 2.1. Study Design

This study is a single-center, open, parallel-group, RCT, conducted at the BioTeSys GmbH, a Clinical Research Organisation in Esslingen, Germany (48° of Northern latitude). The study started on 13 January 2016 (first subject in) and finished on 9 May 2016 (last subject out). This trial was sponsored by Merck Selbstmedikation GmbH (Darmstadt, Germany) and the publication report adheres to the Consolidated Standards of Reporting Trials (CONSORT) 2010 statement [26]. Ethical approval was obtained by the Institutional Review Board (IRB) of the Landesärztekammer Baden-Wurttemberg (ethics committee No.: F-2015-102). The study complies with the Declaration of Helsinki and with the principles of Good Clinical Practice (ICH-GCP). This trial was registered at German Clinical Trials Register (http://www.germanctr.de) (DRKS-ID: DRKS00009770).

### 2.2. Participants

We enrolled apparently healthy women of childbearing age in the study. Main inclusion criteria were female gender, age ≥18 to ≤45 years, body mass index (BMI) 17 to 30 kg/m^2^, good physical and mental health, and no visits to southern countries in the past 30 days and no plans to travel to southern countries during the trial. Main exclusion criteria were any vitamin D supplement intake/prescription in the past two months and during the trial, significant diseases, medication with potential interference with vitamin D metabolism, pregnancy, breast feeding, as well as planning to become pregnant during the study (see Appendix Table A1 for a detailed list of inclusion and exclusion criteria).

Study participants were recruited by advertisements in local newspapers and public notice boards in Esslingen and Stuttgart, and individuals who had already participated in clinical studies at BioTeSys GmbH were informed via e-mail about this trial. All study participants gave written informed consent prior to study inclusion. Study visits were performed at baseline (visit 1), and after 4 weeks (visit 2) and 8 weeks (visit 3) of intervention.

### 2.3. Intervention

Study participants were randomized to receive in a 1:1 ratio either Femibion^®^ 1 (multimicronutrient supplement containing 800 IU vitamin D3; Lot: 488615/090) or Elevit^®^ gynvital (multimicronutrient supplement containing 200 IU vitamin D3; Lot: MA029U8) daily for 8 weeks. Femibion^®^ 1 (Merck Selbstmedikation GmbH, Darmstadt, Germany) was provided by the sponsor and Elevit^®^ gynvital (Bayer Vital GmbH, Leverkusen, Germany) was purchased at wholesale. The nutrition facts of these two multimicronutrient supplements are shown in Table 1.

The originally blistered products of Femibion^®^ 1 (=800 IU group) and Elevit^®^ gynvital (=200 IU group) were repacked into neutral packages and were labelled by a consecutive randomization (participant) number according to a randomization list that was created by the software Randlist.exe, and that was only accessible by the study coordinator. Group allocation according to this randomization list was done in blocks of 10 and was stratified into users and non-users of hormonal contraceptives. All subjects with use of hormonal contraceptives as well as use of hormonal intra-uterine devices were considered “users”. Study participants received their randomization (participant) number according to their consecutive order of study entry.

### 2.4. Primary Outcome Measure and Sample Size Calculation

The primary outcome measure was the between group difference in the increase of 25(OH)D from visit 1 (baseline) to visit 3 (study end after 8 weeks of intervention). Sample size calculation was based on the assumption of normal data distribution with a standard deviation of 21 nmol/L, and a between group difference in the increase of 25(OH)D from baseline to study end of 10.5 nmol/L (expected increase of approximately 1.75 nmol/L per 100 IU vitamin D3 according to a conservative estimate from previous studies) [27]. For a 90% statistical power with a significance level of 5% to detect a significant effect on the primary outcome measure, we calculated a sample size of 86 participants per treatment group. To compensate for a potential dropout rate of 14%, a total sample size of 200 study participants was planned.

### 2.5. Secondary Outcome Measures

Secondary outcome measures were the between group difference in the increase of 25(OH)D from visit 1 (baseline) to visit 2 (after 4 weeks of intervention) and the between group differences in the percentages of participants with 25(OH)D concentrations of ≥50 nmol/L or ≥75 nmol/L at visit 2 and visit 3, respectively. Within group changes in 25(OH)D concentrations from visit 1 to visit 2 and 3 were further outcome measures. Additional pre-defined outcome measures were red blood cell (RBC)-folate, serum folate, and homocysteine, but presenting and discussing data/results on these outcome measures would extend the scope and length of our work and we will therefore publish these findings in a separate manuscript. Pre-specified subgroup analyses were performed for participants with 25(OH)D <50 and ≥50 nmol/L as well as for users and non-users of hormonal contraceptives. During the study intervention, the participants documented any adverse event and concomitant medication in their diaries. 

### 2.6. Measurements

Physical examinations, blood collections, and subject interviews on medical history and medication use were performed at all study visits. Blood pressure and heart rate were measured with an automated device in the sitting position after 5 min at rest. Dietary vitamin D intake was estimated by use of a 3-day food diary protocol that was analysed using EBISPro software (www.ebispro.de) based on nutrient content of the German Nutrient Data Base (Bundeslebensmittelschlüssel, BLS). Compliance was assessed based on dispensed and returned study supplements. In case of lost study supplements, compliance was checked by entries in the participant diaries.

Blood samplings were performed in the morning after an overnight fast of at least 10 h. Participants were instructed to consume a standardized dinner (farm house bread with cream cheese and skinned cucumber) the evening before the study visits, and to take the last study products 24 h prior to agreed blood sampling at visits 2 and 3. Measurements of blood routine parameters were performed at Synlab MVZ Leinfelden-Echterdingen GmbH. Serum 25(OH)D was measured by means of liquid chromatography-tandem mass spectrometry (LC-MS/MS) and the use of the MassChrom^®^ 25-OH-Vitamin D3/D2 kit (Chromsystems GmbH, Gräfelfing, Germany). Inter-assay coefficients of variation were 5.5% (at level 95.8 nmol/L) and 6.7% (at 42.4 nmol/L), respectively. The quality and accuracy of this laboratory method are assured by participation in the vitamin D External Quality Assessment Scheme (DEQAS) on a regular basis. To evaluate the external validity of the 25(OH)D measurement, we compared 30 randomly selected serum samples with another LC–MS/MS method, used by the Vitamin D Research Group at the University College Cork, Ireland [10].

### 2.7. Statistical Analyses

Distribution of efficacy parameters was tested with Shapiro-Wilk test. Because of non-normal distribution, continuous data are presented as medians with interquartile ranges (25th to 75th percentile). Categorical data are shown as percentages. Baseline comparisons between groups were performed by Wilcoxon rank sum test (continuous variables) or Chi square test (categorical data).

Depending on data distribution and statistical assumptions, the analysis of the primary outcome measure was planned to be performed by analysis of co-variance (ANCOVA) with adjustment for baseline 25(OH)D or by Wilcoxon rank sum test [28]. Between group differences for secondary outcome measures were either calculated by ANCOVA or Wilcoxon rank sum test (for continuous variables) or by Chi square or Fishers exact test (for categorical variables). Within group differences were planned to be calculated by repeated measures ANOVA or Friedman test with Dunn’s Multiple Comparison Test (continuous variables) and by the McNemar test (categorical variables). For between and within group differences, a linear mixed model with repeated measurements was applied. 

Analyses were conducted according to the intention to treat (ITT) principle in an ITT population that was defined as participants who met all inclusion criteria and no exclusion criterion, received at least one dose of the study product and had 25(OH)D measurements at baseline and study end. All analyses were performed in this ITT population if not otherwise specified. Within this population, no data imputation for missing values we performed. All randomized participants are part of the safety population to evaluate adverse events.

A *p*-value < 0.05 was considered statistically significant. Statistical analyses were performed by using SAS Version 9.3 (SAS Institute Inc., Cary, NC, USA), SPSS Version 24.0 (IBM SPSS Inc., Chicago, IL, USA) and GraphPad Prism Version 5.04 (GraphPad Software, La Jolla, CA, USA).

## 3. Results

**Figure 1:**
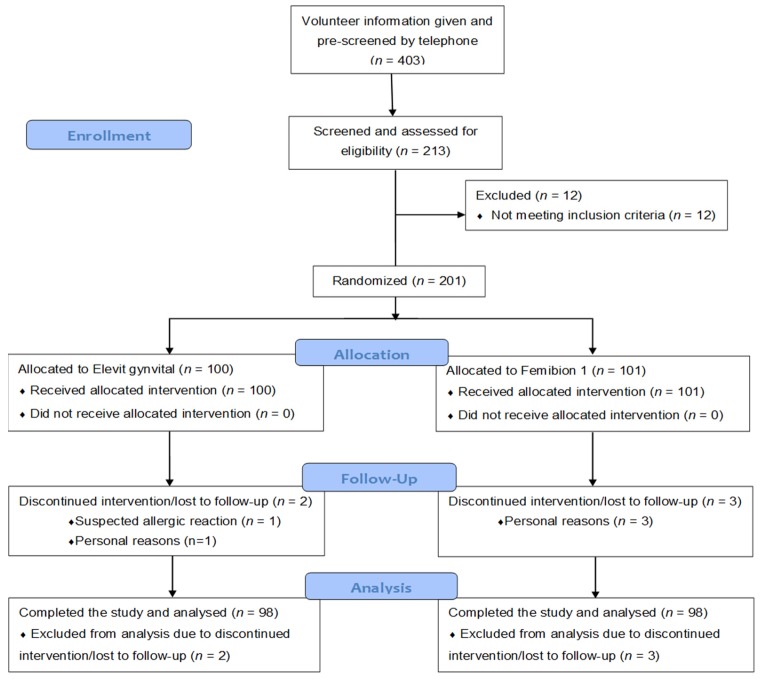
Participant flowchart.

There were 403 individuals who were interested in the study and who were pre-screened by telephone interviews regarding potential eligibility for study inclusion. After this telephone interview, there remained 213 individuals who were still interested in participating in the study and who were judged to be potentially eligible for the trial. These individuals gave written informed consent and were evaluated in a screening visit (visit 1). After excluding 12 individuals (3 due to high liver transaminases, 2 due to personal reasons, and 1 each due to vacation in a southern country, hyperlipidemia, breastfeeding, large intestine resection, low hemoglobin, vitamin D supplementation and indigestion), 201 study participants were randomized. The whole participant flow chart is shown in Figure 1.

There were two drop outs in the 200 IU group (one between visits 1 and 2 due to suspected allergic reaction and one between visits 2 and 3 due to personal reasons) and three drop outs in the 800 IU group (all due to time/personal reasons with two drop outs between visits 1 and 2, and the other one between visits 2 and 3). Only one participant had a major protocol violation due to holidays in Florida, USA. The time on study treatment (mean ± standard deviation) was 55 ± 1 days in both study groups. Compliance (mean ± standard deviation) was 99.3% ± 2.4% in the 200 IU group and 99.6% ± 2.5% in the 800 IU group.

### 3.1. Baseline Characteristics

The 201 randomized participants had a median (interquartile range) age of 25 (22 to 29) years, a BMI of 21.5 (20.1 to 23) kg/m^2^ and 25(OH)D serum levels of 43.7 (31.4 to 59.9) nmol/L. Baseline characteristics of the entire ITT population (*n* = 196) as well as for the two treatment groups are shown in Table 2. In brief, there were no statistically significant differences between the study groups. Concentrations of 25(OH)D below 30, 50, and 75 nmol/L were observed in 23.0%, 60.7%, and 91.8% of the ITT population, respectively. The study population was mainly of Caucasian origin but 6 participants in the 200 IU group and 5 participants in the 800 IU group were judged to be of non-Caucasian origin (not further specified) due to a darker skin colour.

### 3.2. Primary Outcome Analyses

**Figure 2:**
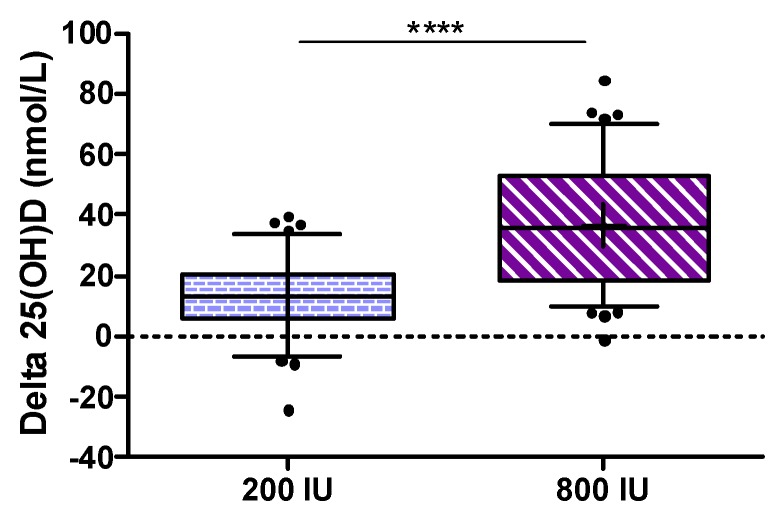
Increase of 25(OH)D serum levels after 8 weeks of supplementation with 200 IU (left) and 800 IU (right) vitamin D3; Box and Whiskers 5–95th percentile; +mean; **** *p* < 0.001 for group comparison with Wilcoxon rank sum test.

The primary endpoint defined as the increase in 25(OH)D from baseline to study end was significantly higher in the 800 IU group compared to the 200 IU group (*p* < 0.001; see Figure 2).

**Figure 3:**
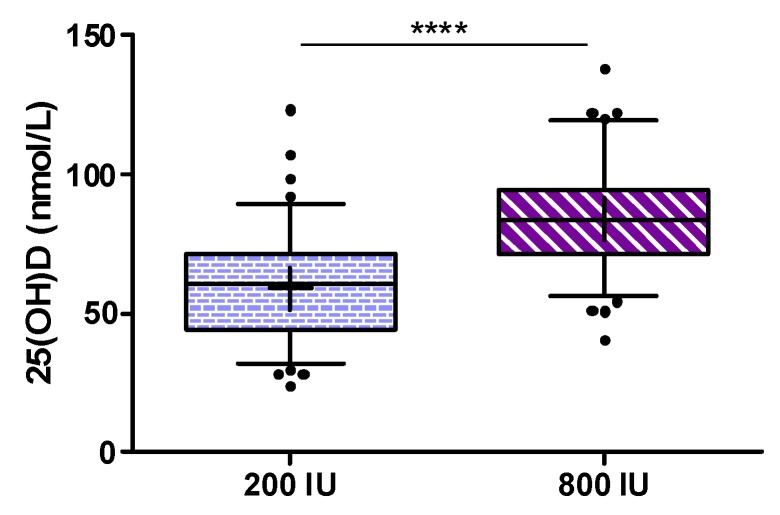
Serum 25(OH)D concentration after 8 weeks of intake of 200 IU (left) and 800 IU (right) vitamin D3; Box and Whiskers 5–95th percentile; +mean; **** *p* < 0.001 for ANCOVA.

Considering some violations of assumptions for the ANCOVA (non-normally distributed data, no homogeneity of variance and no homogeneity of regression slopes) we analyzed (as per protocol) the primary outcome measure by Wilcoxon rank sum test, but an additional analysis by ANCOVA using changes in 25(OH)D with adjustment for baseline 25(OH)D) revealed again a *p*-value of < 0.001. The median (interquartile range) increase in 25(OH)D from baseline to study end was 13.2 (5.9 to 20.7) nmol/L in the 200 IU group and 35.8 (18.2 to 52.8) nmol/L in the 800 IU group. As an additional analysis for the primary outcome, we calculated an ANCOVA for the between group difference in 25(OH)D levels at study end after log transformation of data while adjusting for baseline 25(OH)D concentrations, and observed again a highly significant difference (see Figure 3) with a treatment effect (with 95% confidence interval) for 25(OH)D levels of 25.4 (22.1 to 28.4) nmol/L. 

Additionally to these outcome measures after 8 weeks of interventions, the progression of 25(OH)D concentrations over time and between dosage groups were evaluated in a linear mixed model with repeated measurements. With regard to 25(OH)D, significant effects of intervention (*p* < 0.0001), time (*p* < 0.0001) and a significant interaction of intervention × time (*p* < 0.0001) was observed.

### 3.3. Secondary Outcome Analyses

**Figure 4:**
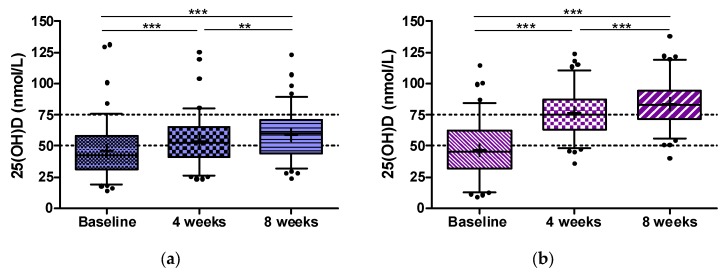
(**a**) Serum 25(OH)D concentration at baseline and after 4 and 8 weeks of supplementation with 200 IU vitamin D3; Box and Whiskers 5–95th percentile; +mean; ** *p* < 0.01; *** *p* < 0.001 for Dunn’s multiple test; (**b**) Serum 25(OH)D concentration at baseline and after 4 and 8 weeks of supplementation with 800 IU vitamin D3; Box and Whiskers 5–95th percentile; +mean; *** *p* < 0.001 for Dunn’s multiple test.

At 4 weeks, there was already a significant between group difference for the increase in 25(OH)D (*p* < 0.001). Distribution and changes of serum 25(OH)D over time within the study groups are shown in Figure 4a,b.

**Figure 5:**
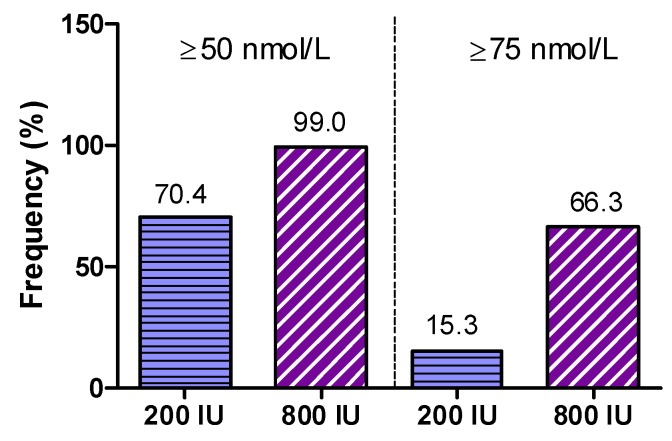
Frequency of subjects showing serum 25(OH)D levels ≥50 nmol/L or ≥75 nmol/L after 8 weeks of supplementation with either 200 IU or 800 IU vitamin D_3_.

In detail, median (interquartile range) 25(OH)D (in nmol/L) at visits 1, 2, and 3 was 42.7 (31.1 to 58.2), 52.4 (41.1 to 65), and 60.3 (43.9 to 71.1) in the 200 IU group, and 45.3 (31.6 to 62.4), 75.1 (62.9 to 87.3), and 83.1 (71.4 to 94.4) in the 800 IU group. Percentages of participants with 25(OH)D concentrations ≥50 and ≥75 nmol/L in both treatment groups at study end are shown in Figure 5.

The main finding was that at study end 70.4% of participants in the 200 IU group and 99% of the 800 IU group had 25(OH)D levels of ≥50 nmol/L (*p* < 0.0001 for Fishers exact test). Levels of ≥75 nmol/L were achieved in 15.3% of participants in the 200 IU group and 66.3% of the 800 IU group (*p* < 0.001 for Fishers exact test). In addition, we show the percentages of individuals per study group for various cut-off levels for 25(OH)D that have been proposed in the scientific literature (Table 3).

### 3.4. Subgroup Analyses

Subgroup analyses in participants with baseline 25(OH)D levels below 50 and ≥50 nmol/L showed that the increase in 25(OH)D concentrations was more pronounced in individuals with lower versus higher 25(OH)D baseline levels (see Table 4). In both intervention groups, there was a significant interaction of baseline 25(OH)D levels with the increase of 25(OH)D from baseline to visit 3 (*p* < 0.01 for both groups with ANCOVA).

Significantly higher median (with interquartile range) 25(OH)D baseline levels were observed in users of hormonal contraceptives (48.3 (33.1 to 62.5) nmol/L; *n* = 118) when compared to non-users (39.1 (25.5 to 52.5) nmol/L; *n* = 78). In subgroups of users and non-users of hormonal contraceptives, we detected not only higher 25(OH)D baseline levels, but also a more pronounced response to vitamin D supplementation in users of hormonal contraceptives (see Table 5). The increase in 25(OH)D from baseline to visit 3 was significantly higher in users versus non-users in the 800 IU group (*p* < 0.05) but not in the 200 IU group.

Due to absence of effective sunlight exposure during winter, a decrease of 25(OH)D levels over winter time was expected and confirmed within the study. Baseline 25(OH)D levels of subjects enrolled in January (47.9 (35.8–59.9) nmol/L; *n* = 61) were significantly higher in comparison to subjects starting in February (39.4 (29.2–58.4; *n* = 75), *p* = 0.029. In March (*n* = 60), median baseline concentrations of 25(OH)D were 44.4 (29.3–60.8) nmol/L and not significantly different compared to February (*p* = 0.352). 

To estimate a possible impact of sunlight-induced vitamin D synthesis in the skin in participants who finished the trial in April and May, we decided (after finishing the trial) to perform subgroup-analyses in all participants who finished the trial by the end of April (*n* = 160) and March (*n* = 72).

In participants who finished the trial by the end of April, 68.8% in the 200 IU group and 98.8% in the 800 IU group achieved 25(OH)D levels of at least 50 nmol/L (*p* < 0.001 for between group difference), and 13.8% in the 200 IU group and 66.3% in the 800 IU group achieved 25(OH)D levels of at least 75 nmol/L (*p* < 0.001 for between group difference). In that group, 25(OH)D levels at study end were 59.7 (43.7 to 69.6) nmol/L in the 200 IU group and 81.0 (71.7 to 91.7) nmol/l in the 800 IU group (*p* < 0.001 for ANCOVA).

In participants who finished the trial by the end of March, 59.5% in the 200 IU group and 97.1% in the 800 IU group achieved 25(OH)D levels of at least 50 nmol/L (*p* < 0.001), and 10.8% in the 200 IU group and 60.0% in the 800 IU group achieved 25(OH)D levels of at least 75 nmol/L (*p* < 0.001). In that group, 25(OH)D levels at study end were 55.7 (39.4 to 68.0) nmol/L in the 200 IU group and 78.4 (70.6 to 91.4) nmol/L in the 800 IU group (*p* < 0.001 for ANCOVA).

### 3.5. External Comparison of 25(OH)D LC-MS/MS Assays

**Figure A1:**
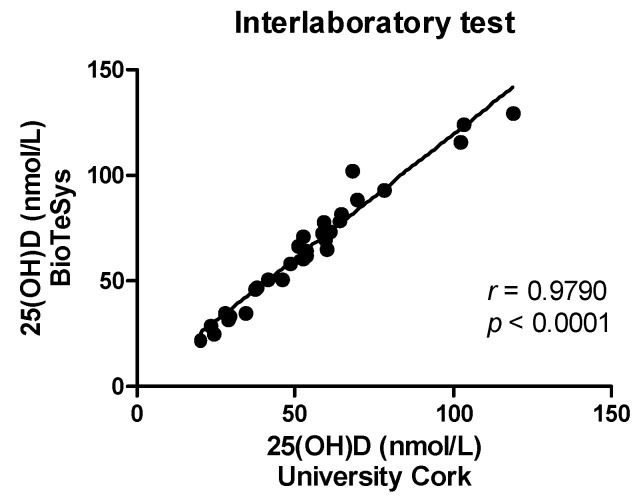
Pearson correlation between 25(OH)D levels measured by Chromsystems and University of Cork LC–MS/MS methods.

In 30 randomly selected samples (10 of each visit), 25(OH)D levels were measured with a validated tandem MS method used by the Vitamin D Research Group at University College Cork, Ireland [10]. Pearson correlation coefficient between both 25(OH)D methods was excellent with *r* = 0.979 (*p* < 0.001). There was, however, a systematic bias towards lower 25(OH)D levels measured in Cork: mean (standard deviation) for 25(OH)D was 54.3 (23.8) nmol/L measured by the method in Cork and 65.4 (28.6) nmol/L measured by the Chromsystems assay (see Appendix Table A2 and Figure A1). We did not re-analyze our whole study based on 25(OH)D data imputation according to the Cork method because we had only 30 available values and we cannot be absolutely certain that the Cork method reveals the true 25(OH)D levels (e.g., potential impact of shipping/storage etc.). However, considering that there was a systematic bias towards approximately 20% higher levels with the Chromsystems assay, we evaluated the percentages of individuals with 25(OH)D levels ≥60 (50 plus 10) nmol/L at all study visits, and observed that at study end the vast majority in the 800 IU group was at or above this threshold, whereas half of the participants in the 200 IU group did not reach 60 nmol/L at study end (see Table 3). The difference between the groups remained highly significant when using 60 nmol/L as a threshold (*p* < 0.001).

### 3.6. Safety Data

In summary, 501 adverse events (AE) were reported by 170 subjects during the trial. There were no serious adverse events (including no death). There were also no apparent differences in reported adverse events between the study groups. Moreover, no participant became pregnant during the study. From the 501 reported AE, only 10 were classified as intolerance reactions to the study medication. The reported intolerance reactions did not raise any new safety concerns. 

## 4. Discussion

In this randomized trial, we have shown that in German women of childbearing age, a multimicronutrient supplement containing 800 IU vitamin D3 was superior compared to a supplement containing 200 IU vitamin D3 in raising 25(OH)D levels and achieving a level of at least 50 nmol/L during wintertime. Participants with low 25(OH)D had a more pronounced increase in 25(OH)D and users of hormonal contraceptives had higher baseline levels and a stronger increase in 25(OH)D levels after vitamin D supplementation when compared to non-users.

Our findings support the approach of the DACH Nutrition Society to raise the intake recommendations for vitamin D from 200 to 800 IU per day in order to achieve a 25(OH)D level of at least 50 nmol/L in individuals with no endogenous vitamin D synthesis, a condition that is usually present during winter in Northern European countries. This recommendation was mainly based on studies performed in Ireland suggesting that 800 IU of vitamin D per day are sufficient to meet the dietary requirements [12,13]. Our trial is, to the best of our knowledge, the first randomized trial to compare the “old”, i.e., 200 IU per day, versus the “new”, i.e., 800 IU per day, dietary vitamin D intake recommendation in the general population in Germany. The only other study that specifically addressed this question during winter in Germany was a placebo controlled trial of 105 participants (age range: 20 to 71 years; 67% females) [14]. In that randomized controlled trials (RCT) by Lehmann et al., only participants with 25(OH)D concentrations below 75 nmol/L were included and randomly allocated to 800 IU vitamin D3 per day or placebo [14]. After 12 weeks on 800 IU of vitamin D3 per day, 94% of the study participants achieved a 25(OH)D serum concentration of at least 50 nmol/L. Our study significantly extends the findings of the RCT by Lehman et al. by including about twice as many study participants, by not restricting our study to individuals with low 25(OH)D concentrations, and by providing data on the efficacy of 200 IU vitamin D per day, a dose that is still used in many vitamin D supplements. Furthermore, we exclusively included women of childbearing age, in whom the vitamin D status is of particular importance because in the case of pregnancy, the 25(OH)D level of the developing fetus as well as of the newborn child is totally dependent on maternal 25(OH)D levels [1,20,29]. Considering that a German study in pregnant women reported that 25(OH)D levels in winter are below 50 nmol/L in 98% of maternal and 94% of cord blood levels, there exists an urgent need to improve vitamin D status in such populations [29]. This high prevalence of vitamin D deficiency in pregnant women is of concern, because low 25(OH)D levels have been associated with various adverse pregnancy outcomes, and there is accumulating evidence from RCTs that vitamin D supplementation during pregnancy is safe and may even improve outcomes such as bone mineral content and density (if vitamin D is supplemented in winter), pre-eclampsia, low birth weight, and preterm birth [1,20,21,22,23]. Our findings in women of childbearing age are also in line with several other reports on a high prevalence of vitamin D deficiency in the general population, and it is of particular concern that at baseline, 60.7% of our study population had 25(OH)D levels below 50 nmol/L, and even 23% had 25(OH)D levels below 30 nmol/L. Since many women of childbearing age are taking supplements prior to conception, we believe that our study findings strongly argue for a vitamin D supplemental dose of 800 IU per day. In this context, it noteworthy that a daily vitamin D dose of 800 IU is the dietary intake recommendation (under conditions of no endogenous sun exposure) for non-pregnant as well as pregnant women, with some studies showing that vitamin D sufficiency is associated with reduced risk of miscarriage, an intriguing observation that has to be further evaluated in RCTs [30].

When interpreting the results of the present study, we have to take into account the overall vitamin D intake, i.e., the study supplement plus the dietary vitamin D intake. According to our data, the median vitamin D intake was only about 1.8 µg (72 IU) vitamin D per day. Although, we may have slightly underestimated the dietary vitamin D intake in our trial, there are data from a national survey in Germany indicating that in women, the median dietary vitamin D intake is only approximately 100 IU per day [31]. Therefore, the actual vitamin D intake that we compared in our trial was approximately 300 versus 900 IU (dietary plus supplemental) vitamin D per day [31]. Uncertainties regarding the true vitamin D content of the diet as well as of the supplement remain, but this is a general limitation of all studies in this field. Nevertheless, despite uncertainties and limitations of true vitamin D intakes, our study results strongly argue that a vitamin D intake below 800 IU per day may not be sufficient to ensure that the vast majority of the population achieves a 25(OH)D level of at least 50 nmol/L during winter in Germany. These data will be relevant for future vitamin D recommendations. In this context, the European Food Safety Authority (EFSA) has released new dietary reference values for vitamin D [32] in October 2016. For adults, the adequate intake (AI) for vitamin D was set at 600 IU per day, assuming that this intake is sufficient to achieve 25(OH)D levels of near or above the target of 50 nmol/L in the majority of the population [32].

The higher increase in 25(OH)D levels in individuals with lower versus higher baseline concentrations confirms previous investigations [32]. Interestingly, users of hormonal contraceptives have higher 25(OH)D levels when compared to non-users. This result is in line with other cross-sectional studies. Harmon et al reported in 1662 African-American women aged 23 to 34 years, that users of estrogen-containing contraceptives had 20% higher 25(OH)D levels when compared to non-users [25]. It remains to be clarified in detail how hormonal contraceptives increase 25(OH)D levels, but previous studies suggest that increased vitamin D binding protein (DBP) levels and an effect on vitamin D metabolizing enzymes (e.g., higher 25-hydroxylation in the liver) may be underlying mechanisms for the effect of hormonal contraceptives on 25(OH)D levels [24,25]. Moreover, the stronger increase in 25(OH)D levels after vitamin D supplementation in users as compared to non-users of hormonal contraceptive users further extends our knowledge on this issue and confirms study results by Nelson et al. [24]. We are of the opinion that this has clinical implications and we agree with Harmon et al. who concluded that clinical testing of 25(OH)D status should take into account recent contraceptive use. Women who are planning a pregnancy may be at particular high risk of vitamin D deficiency, because 25(OH)D levels may markedly drop after cessation of hormonal contraceptives [25]. The pathophysiologic effects of this increase in 25(OH)D with use of hormonal contraceptives with regard to its impact on bone and mineral metabolism remains to be further elucidated. It is interesting to note that a similar effect of hormonal contraceptives on total cortisol levels is considered to be without major clinical significance because the increase in cortisol is mainly driven by higher cortisol binding globulin levels with a subsequent increase in total cortisol levels, though not in the free and biologically relevant cortisol concentration [33].

One limitation of our trial is that our results cannot be uncritically extrapolated to a national representative population. Although we recruited participants that likewise represent a good footprint of the general population of women of childbearing age in Germany, we cannot rule out some sort of selection bias. Furthermore, the duration of the trial with 8 weeks may be considered as too short to achieve a steady state in 25(OH)D levels. In this context, we want to note that our findings, along with previous RCT results, suggest that a saturation of 25(OH)D levels has already been reached after 8 weeks of treatment [14,34,35]. Another drawback of our trial may be that it was not strictly performed in wintertime so that sunlight-induced vitamin D synthesis may have significantly increased 25(OH)D levels in participants finishing the trial in April or May. However, subgroup analyses in individuals finishing the trial by the end of March or April revealed similar results as in the overall cohort. A further limitation of our work is the result of the 25(OH)D assay comparison, with a systematic bias towards lower levels measured by the method in Cork. Both methods are well validated but a plausible explanation for the higher 25(OH)D concentrations by the Chromsystems method may be the missing separation of additional vitamin D metabolites such as the 3-epimer [36]. We did not perform 25(OH)D data imputation and re-analyses for the whole study according to the 30 values obtained by the method in Cork, but we want to stress that even when assuming a true overestimation of 25(OH)D by the Chromsystems assay with 20% and a therefore desired 25(OH)D level of at least 60 (50 + 10) nmol/L, the vast majority of the women achieves this level with 800 IU vitamin D per day. Therefore, we consider this issue to not violate our main results on the treatment effect on raising 25(OH)D levels. A further limitation of our trial is that we have not measured other parameters of bone and mineral metabolism such as parathyroid hormone. Our trial is, however, novel because, to the best of our knowledge, no previous RCT in the general population in wintertime has, with a comparable sample size, specifically addressed the effect of vitamin D supplementation with 200 IU versus 800 IU per day in achieving the recommended 25(OH)D level of at least 50 nmol/L.

## 5. Conclusions

In conclusion, we have shown in this randomized trial in women of childbearing age in Germany that supplementing 800 IU vitamin D3 per day was safe and sufficient to achieve 25(OH)D levels of at least 50 nmol/L in almost all study participants within 8 weeks, whereas supplementing 200 IU per day was insufficient. These data along with findings on higher 25(OH)D levels at baseline and after vitamin D3 supplementation in users versus non-users of hormonal contraceptives significantly add to the knowledge on dietary vitamin D requirements in Germany.

## Figures and Tables

**Table 1:** Nutrient labeling of Elevit^®^ gynvital and Femibion^®^ 1.

Ingredients	Elevit^®^ Gynvital	Femibion^®^ 1
Folate	400 µg Folate (Folic acid/l-Methylfolate (1:1))	800 µg Folate (Folic acid/l-Methylfolate (1:1))
Vitamin B1	1.4 mg	1.2 mg
Vitamin B2	1.4 mg	1.6 mg
Vitamin B6	1.9 mg	1.9 mg
Vitamin B12	2.6 µg	3.5 µg
Biotin	30 µg	60 µg
Niacin	18 mg	15 mg
Pantothenic acid	6 mg	6 mg
Vitamin C	85 mg	110 mg
Vitamin E	10 mg	13 mg
Vitamin A	770 µg	-
Vitamin D3	5 µg/200 IU	20 µg/800 IU
Iodine	150 µg	150 µg
Copper	1000 µg	-
Iron	14 mg	-
Magnesium	57 mg	-
Selenium	60 µg	-
Zinc	10 mg	-
Omega-3-fatty acids	200 mg	-

**Table 2:** Baseline characteristics of participants who completed the trial (=intention to treat population).

Characteristics	All Participants	200 IU Group	800 IU Group	*p*-Value
Number	196	98	98	
Age (years)	25 (22–29)	26 (23–29)	25 (22–29.3)	0.600
Body mass index (kg/m^2^)	21.5 (20.1–23.0)	21.4 (20.0–23.0)	21.5 (20.2–22.9)	0.592
25-hydroxyvitamin D (nmol/L)	43.8 (31.4–59.9)	42.7 (31.1–58.2)	45.3 (31.6–62.4)	0.559
Vitamin D intake (µg/day) *	1.76 (1.20–2.51)	1.71 (1.1–2.5)	1.78 (1.26–2.67)	0.251
Hormonal contraceptives (%)	60.2	62.2	58.2	0.559
Active smoker (%)	16.3	19.4	13.3	0.246
Systolic blood pressure (mm Hg)	116 (108–124)	115 (106–124)	118 (111–124)	0.161
Distolic blood pressure (mm Hg)	74 (68–80)	74 (69–80)	74 (68–80)	0.565
Heart rate (beats/min)	73 (67–81)	72 (67–80)	75 (67–83)	0.403

Data are presented as median (interquartile range) or as percentages; *p*-value for Wilcoxon rank sum test or Chi square test of 200 IU group versus 800 IU group; * assessed by a 3 days food diary protocol.

**Table 3:** Distribution of participants according to different cut-off values for 25-hydroxyvitamin D.

25(OH)D in nmol/L	200 IU Group			800 IU Group		
	Visit 1 (*n* = 100)	Visit 2 (*n* = 99)	Visit 3 (*n* = 98)	Visit 1 (*n* = 101)	Visit 2 (*n* = 99)	Visit 3 (*n* = 98)
<25	13.0 *	2.0	1.0	17.8	0.0	0.0
<30	25.0	6.1	4.1	21.8	0.0	0.0
<40	45.0	22.2	19.4	44.6	1.0	0.0
<50	64.0	44.4	29.6	58.4	8.1	1.0
≥50	36.0	55.6	70.4	41.6	91.9	99.0
≥60	21.0	39.4	51.0	26.7	83.8	88.8
≥75	6.0	7.1	15.3	10.9	49.5	66.3
≥125	2.0	1.0	0.0	0.0	0.0	1.0

* Percentages of participants are shown.

**Table 4:** 25(OH)D serum concentrations in the treatment groups at visits 1, 2 and 3 stratified according to baseline 25(OH)D concentrations < and ≥ 50 nmol/L.

25(OH)D in nmol/L	25(OH)D < 50 nmol/L						25(OH)D ≥ 50 nmol/L					
	200 IU Group			800 IU Group			200 IU Group			800 IU Group		
	Visit 1	Visit 2	Visit 3	Visit 1	Visit 2	Visit 3	Visit 1	Visit 2	Visit 3	Visit 1	Visit 2	Visit 3
Number	62	62	62	57	57	57	36	36	36	41	41	41
Minimum	14.0	23.7	24.0	9.0	35.9	40.2	50.4	55.9	52.4	50.4	56.4	64.1
25% Percentile	25.9	34.9	39.4	24.0	60.0	66.8	131.5	125.5	123.3	57.5	72.0	75.4
Median	33.3	43.6	51.3	33.4	68.9	78.4	56.2	62.1	66.5	64.9	85.1	87.4
75% Percentile	41.9	51.5	61.2	39.6	78.9	90.1	61.0	68.0	72.1	75.4	101.3	107.2
Maximum	49.9	66.9	80.6	49.9	106.3	122.1	72.3	74.6	81.4	115.1	124.1	138.3

**Table 5:** 25(OH)D serum concentrations in the treatment groups at visits 1, 2 and 3 stratified according to “no use” and “use” of hormonal contraceptives.

25(OH)D in nmol/L	No Use of Hormonal Contraceptives						Users of Hormonal Contraceptives					
	200 IU Group			800 IU Group			200 IU Group			800 IU Group		
	Visit 1	Visit 2	Visit 3	Visit 1	Visit 2	Visit 3	Visit 1	Visit 2	Visit 3	Visit 1	Visit 2	Visit 3
Number	37	37	37	41	41	41	61	61	61	57	57	57
Minimum	14.0	23.7	28.2	9.0	45.4	50.9	19.5	25.7	24	12.5	35.9	40.2
25% Percentile	28.2	34.9	39.3	24	58.5	64.1	31.8	44.9	54.2	33.3	75.3	79.9
Median	38.2	45.4	50.2	39.2	64.1	71.9	47.9	56.7	64.1	48.7	83.9	91.1
75% Percentile	48.2	61.2	61.7	54.3	70.6	81.0	60.0	68.0	72.9	69.0	95.6	104.6
Maximum	75.6	74.6	80.6	73.6	89.6	94.1	131.5	125.5	123.3	115.1	124.1	138.3

## Appendix A

**Table A1:** Inclusion and exclusion criteria for the study.

Inclusion Criteria
(1) Subject is able and willing to sign the Informed Consent Form prior to screening evaluations
(2) Healthy volunteers: Subject is in good physical and mental health as established by medical history, physical examination, vital signs, and results of biochemistry and haematology
(3) Women of childbearing age
(4) Age ≥18 and ≤45 years
(5) Body mass index: 17–30 kg/m^2^
(6) No travel into southern countries in the past 30 days
(7) No plans to travel into southern countries during the study
(8) Able and willing to follow the study protocol procedures
**Exclusion Criteria**
(1) Clinically relevant abnormal vital signs, physical findings, or laboratory values
(2) Taking any medication interfering with vitamin D metabolism, e.g., cardiac glycosides, barbiturates, anti-tuberculosis drugs (rifampicin, isoniazid), cholestyramine, glucocorticoids (except inhalative), benzothiadiazine derivatives, anti-convulsants, parathyroid hormone or parathyroid hormone derivatives in the past 6 months
(3) Vitamin D prescription
(4) Intake of the antibiotics Pyrimethamin or Trimethoprim two weeks prior to visit 1 as both anti-biotics interfere with the folate metabolism
(5) Taking other dietary supplements/medication with folate and/or vitamin D in the past 2 months
(6) Concomitant use of cod liver oil or intake during the past 2 months
(7) Because of thyroid disorder intake of iodine contraindicated
(8) Sarcoidosis or any other granuloma-forming inflammatory disease
(9) Osteoporosis, osteomalacia
(10) History of diabetes, stroke, cardiovascular disease and cancer
(11) Fat malabsorption (Crohn’s or celiac disease, cystic fibrosis)
(12) Gastrointestinal diseases/conditions (colitis ulcerosa, Crohn’s disease, irritable bowel syndrome, inflammatory bowel disease, peptic ulcers) hindering vitamin D absorption
(13) Liver or renal disease (including alcoholism)
(14) Pancreatic insufficiency (disturbed fat metabolism)
(15) Kidney stones or history of hypercalcaemia and hypercalciuria
(16) Psychological disorders
(17) Calcium metabolism disorders
(18) Hypo- and Hyperparathyroidism
(19) Participants anticipating a change in their lifestyle or physical activity levels during the study.
(20) Subjects with history of drug, alcohol or other substances abuse, or other factors limiting their ability to co-operate
(21) Known hypersensitivity to the study preparation or to single ingredients (e.g., allergy to crustaceans or fish)
(22) Pregnant subject or subject planning to become pregnant during the study; breast-feeding subject.
(23) Known HIV infection
(24) Known acute or chronic hepatitis B and C infection
(25) Blood donation within 4 weeks prior to visit 1 or during the study
(26) Subject involved in any clinical or food study 4 weeks prior to visit 1

**Table A2:** Assay comparison for 25(OH)D measurements (in nmol/L).

	University Cork	Chromsystems
Number of values	30	30
Minimum	20.0	22.2
25th percentile	36.8	43.5
Median	53.0	64.9
75th percentile	64.2	79.3
Maximum	119.0	129.8
Mean	54.3	65.4
Standard deviation	23.8	28.6
